# Drug-Coated Balloon (Optilume®) for the Management of Bulbar Urethral Stricture, Our Experience

**DOI:** 10.5152/tud.2025.25020

**Published:** 2025-06-04

**Authors:** Ibrahim Alnadhari, Muammer Alshrani, Asim Alhattami, Walid Shanaa, Omar Ali, Osama Abdeljaleel, Hana J. Abukhadijah, Ahmad Shamsodini

**Affiliations:** 1 Department of Surgery, Urology Section, Al Wakra Hospital, Hamad Medical Corporation, Doha, Qatar; 2 Department of Surgery, Qatar University, Doha, Qatar; 3 Department of Academic Health System, Hamad Medical Corporation, Doha, Qatar

**Keywords:** Urethra, urethral stricture, recurrence, paclitaxel

## Abstract

**Objective:**

The drug (paclitaxel)-coated balloon (DCB) Optilume® is designed to dilate the urethral lumen via balloon dilation while also promoting long-term urethral patency through the targeted and circumferential delivery of paclitaxel. As an antimitotic agent, paclitaxel functions by inhibiting cellular proliferation and migration. The objective of this study is to assess the safety and efficacy of this DCB in managing bulbar urethral strictures.

**Methods:**

This study is a retrospective study, involving 19 patients who underwent treatment with DCB for bulbar strictures. Patients’ characteristics, preoperative and postoperative maximum flow rates (Qmax), and the duration until recurrence were recorded. Postoperative complications were also noted.

**Results:**

Success was achieved in 15 out of 19 patients (78.9%) with a median follow-up period of 352 days. The mean maximum urine flow rate preoperatively was 5 mL/s (with a range of 4.00-6.50 mL/s). Subsequent mean flow rates at 3 months, 6 months, 12 months, and 24 months post-treatment were 32 mL/s, 32 mL/s, 24 mL/s, and 20 mL/s, respectively. Notably, there was no recurrence among the 7 patients without previous surgical interventions (47%, *P*-value .01). The mean duration of stricture-free survival following surgery was 648 days (approximately 21.6 months) (95% CI 500.4-700.2). Importantly, no complications were reported throughout the study.

**Conclusion:**

The use of DCB for managing bulbar urethral stricture demonstrated a success rate of 78.9%. Patients without a history of urethral surgery exhibited significantly improved treatment outcomes. Furthermore, the study reported no significant complications associated with the use of DCB.

**Main Points**

- Drug (paclitaxel)-coated balloon showed a good success rate with a lower recurrence rate.
- None of the patients with no prior urethral surgery experienced a recurrence, which suggests that patients undergoing initial urethral stricture surgery with DCB might benefit more.
- Drug (paclitaxel)-coated balloon showed a good safety profile with no reported complications.

## Introduction

Urethral stricture poses a considerable challenge due to its elevated recurrence rate, particularly following prior surgical procedures. Studies indicate that repeated endoscopic interventions are associated with even higher recurrence rates.^1^,^2^ The success rate following the first internal urethrotomy is estimated to be between 55% and 60%; however, this rate diminishes with subsequent procedures. Specifically, the success rate ranges from 0% to 40% at 48 months and reaches 0% at 24 months after the second and third urethrotomy, respectively.^1^ In the context of managing recurrent strictures post-urethroplasty, endoscopic treatment has been shown to yield a recurrence rate varying from 10% to 50%.^3^,^4^

To address the high recurrence rate after minimally invasive treatment of urethral strictures, various adjunctive therapies have been explored. These include intralesional injections of steroids, mitomycin C, captopril, platelet-rich plasma, and hyaluronidase; drug-coated catheters (e.g., those with steroids); drug-coated balloons (DCBs) (e.g., with paclitaxel); brachytherapy; oral steroids; and tamoxifen.^5^,^6^

Urethral stents, whether temporary or permanent, have been increasingly utilized for the prevention of recurrence and the maintenance of urethral patency owing to their minimally invasive nature.^7^^-^^11^

Recently, several studies have explored the application of targeted antifibrotic agent injections as a supplementary treatment to endoscopic procedures, with the objective of preventing or minimizing the formation of scar tissue.^12^ The DCB Optilume® (Urotronic, Inc., Plymouth, Minn, USA) represents the first DCB specifically developed for the treatment of male anterior urethral strictures. This innovative technology aims to provide immediate symptomatic relief by enlarging the urethral lumen through balloon dilation, while also promoting long-term urethral patency via the circumferential and localized delivery of paclitaxel. Paclitaxel is an antimitotic agent that inhibits cell proliferation and migration and has been widely used in cardiovascular procedures to prevent restenosis following angioplasty.^13^,^14^ ROBUST I trial results, which evaluated the drug (paclitaxel)-DCB in men with recurrent urethral strictures up to 2 cm in length.^15^ It showed that anatomic success was achieved in 70% at 1 year based on the ability to pass a 16F flexible cystoscope or 14F Foley catheter.^15^ Functional success occurred in 70% (32/46) at 2 years, defined as International Prostate Symptom Score (IPSS) improvement ≥50% in the absence of retreatment need.^16^

Our study aims to evaluate the efficacy and safety of the DCB for the treatment of bulbar urethral stricture.

## Material and Methods

### Participants

This study is retrospective in nature, involving 19 patients who received treatment with a DCB (paclitaxel) for bulbar strictures. The study encompassed cases from August 2022 to February 2024. Inclusion criteria were adult male patients (≥18 years old) diagnosed with symptomatic bulbar urethral strictures confirmed by retrograde urethrography (RUG), with or without flexible cystoscopy. Patients with recurrent strictures following one or more endoscopic interventions and those who failed urethroplasty were included. Exclusion criteria included strictures in the penile or posterior urethra, history of pelvic radiation, prior hypospadias repair, active urinary tract infections, or severe comorbidities precluding surgical intervention. Data collected included patient age, comorbidities, history of previous urethral surgeries, and characteristics of the stricture such as its location, length, and underlying cause. Both preoperative and postoperative maximum flow rates (Qmax) were recorded, along with the time to recurrence. Patients were followed postoperatively with uroflowmetry at 3-month intervals for the first year, then every 6 months. Additionally, any postoperative complications were noted. If the patient showed a decrease in urine flow, further evaluation via flexible cystoscopy or urethrogram was conducted. Recurrence was diagnosed if the Qmax <15 mL/s and flexible cystoscope 16 F did not pass or if there was the presence of severe narrowing in the urethrogram. The research received approval from the Hamad Medical Corporation committee (MRC-01-24-234 Date: 04 June 2024) and was conducted in compliance with the Helsinki Declaration.Informed consent was waived by the Medical Research Center Ethics Committee due to the retrospective nature of the study.

### Surgical Technique

Prophylactic antibiotic (cefuroxime 1.5 mg single dose) was administered at anesthesia induction. The procedure was performed under spinal or general anesthesia. A 0.035-inch hydrophilic guidewire was introduced into the urethra and advanced into the bladder under direct vision. The stricture was then dilated using sequential dilators or an optical urethrotome to an appropriate size for balloon insertion. A paclitaxel-coated DCB (Optilume®) was introduced over the guidewire and inflated to the required pressure for 90 s, ensuring adequate drug delivery to the stricture site. After deflation, the balloon was removed, and a 12-14 F urethral catheter was placed for 2-3 days. Postoperatively, patients were monitored using uroflowmetry.

### Statistical Analysis

Categorical variables were presented as frequencies and proportions, and for continuous variables, means with SDs were reported. The statistical significance of differences within categorical and continuous variables was calculated using chi-square and Kruskal–Wallis tests, respectively. The authors used the Kaplan–Meier method to evaluate stricture-free survival. SPSS software was used for statistical analyses, with a 2-sided significance level set at *P* < .05.

## Results

The average age of the participants was 50 ± 14.43 years, while the mean length of the stricture measured 3 ± 1.43 cm. Among the cohort, 12 patients (63%) had a history of previous urethral surgeries, 8 patients (42%) had previous endoscopic surgery, and 4 patients (21%) had undergone 2 or more such procedures. Out of the total number of patients, 4 (21%) experienced a failed urethroplasty. The etiologies of the strictures were categorized as iatrogenic (32%), idiopathic (47%), infectious (16%), and traumatic (5%). The variables and their correlations are presented in Table 1.

**Figure 1.**
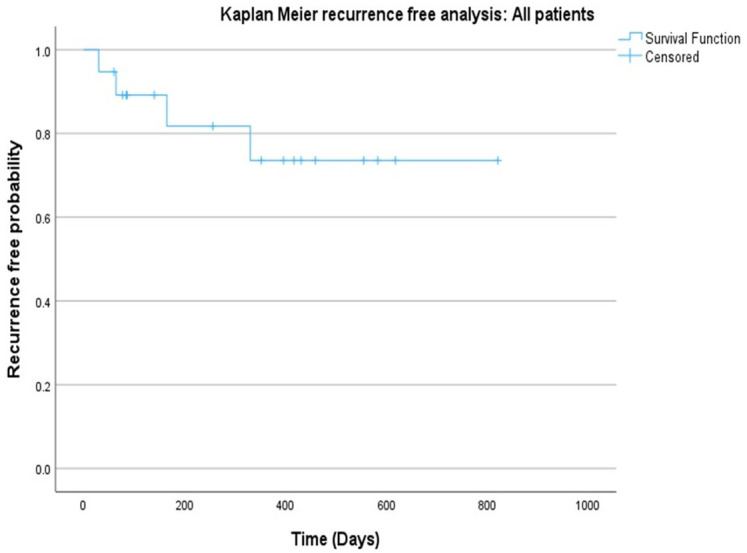
Kaplan-Meier curve for free from recurrence for all patients.

**Figure 2:**
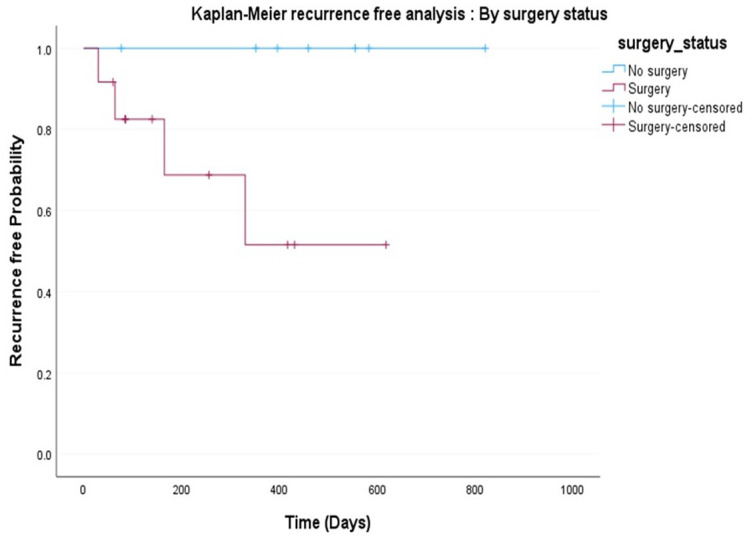
Kaplan-Meier curve for free-from-recurrence analysis between the group with no previous surgery and the group with previous surgery.

Success was noted in 15 out of 19 patients, resulting in a success rate of 78.9%, with a median follow-up period of 352 days (ranging from 90 to 583 days). The mean maximum urine flow rates prior to the application of the DCB were recorded at 5 mL/s (with a range of 4.00-6.50 mL/s). After 3 months, 6 months, 12 months, and 24 months, the mean flow rates were observed to be 32 mL/s, 32 mL/s, 24 mL/s, and 20 mL/s, respectively. Notably, no recurrences were reported among the 7 patients who had not undergone prior surgery (47%, *P*-value .01). The study reported no complications. The mean duration of stricture-free survival was 648 days (approximately 21.6 months) (95% CI 500.4-700.2). As illustrated in Figure 1, the analysis indicated that no recurrences occurred in the group without prior surgery, whereas 4 recurrences were noted in the surgical group, suggesting an increased risk of recurrence for patients who had previously undergone surgery. In the surgical cohort, 51.6% of patients experienced at least 330 days without recurrence (Figure 2). The difference between the 2 groups approached statistical significance with a *P*-value of .056, indicating a trend that may have clinical relevance and necessitating further exploration in a larger population, despite not achieving conventional statistical significance (*P* < .05).

## Discussion

The overall clinical success of DCB in this study was 78.9% at a median follow-up of 352 days. The ROBUST I trial evaluated the safety and efficacy of the DCB for the treatment of male anterior bulbar urethral strictures. At 3 years, functional success occurred in 67% with no treatment-related serious adverse events.^15^ In the ROBUST III randomized controlled study, Elliott et al.^17^ reported a success rate of 83%. In this study, none of the 7 patients with no prior surgery experienced a recurrence (47%, *P-*value .01). The likelihood of stricture recurrence is influenced by the number of prior surgical interventions as well as the specific characteristics of the stricture, including its location, length, caliber, number, and underlying cause. The evaluation of the success of urethral stricture surgery encompasses anatomical success, functional success, and the absence of the need for further surgical procedures. In this assessment, the authors incorporated functional evaluations, such as uroflowmetry, alongside anatomical assessments through ascending and micturating urethrograms and diagnostic cystoscopy to verify any recurrence. The difference in recurrence-free survival between the patients without previous surgery and patients with previous surgery was marginally significant, with a *P*-value of .056, indicating a potential clinical significance trend that warrants further investigation in a larger sample size despite not reaching conventional statistical significance (*P* < .05). This suggests that patients undergoing initial urethral stricture surgery with DCB might benefit more, although the DCB has also demonstrated excellent results for recurrent bulbar urethral strictures. Stricture length and the number of previous urethral surgeries were shown as predictors for recurrence.^1^,^18^ But in this study, there was no significant clinical correlation. The small sample sizes within the subgroups can be the reason or other reasons related to the DCB itself.

This study is a retrospective analysis, which comes with inherent limitations, such as the potential for missing patients and reporting bias. Larger-scale studies could provide more precise outcome definitions with a greater number of participants.

The paclitaxel-coated DCB achieved a success rate of 78.9% for the treatment of bulbar urethral stricture at a median follow-up of 352 days. Patients without prior urethral surgery exhibited significantly better outcomes. Additionally, no significant complications were reported with the use of the DCB.

## Figures and Tables

**Table 1.** Variables and Correlation Table

	Total	Recurrence		*P*
		Yes	No	
	N = 19	N = 4	N = 15	
**Age/years**	50 (14.43)	64 (17.63)	46 (11.31)	.022
**Comorbidities**				.090
0	68% (13)	50% (2)	73% (11)	
1	16% (3)	0% (0)	20% (3)	
≥2	16% (3)	50% (2)	7% (1)	
**Etiology of previous stricture**				.20
Iatrogenic	32% (6)	75% (3)	20% (3)	
Infectious	16% (3)	0% (0)	20% (3)	
Traumatic	5% (1)	0% (0)	7% (1)	
Idiopathic	47% (9)	25% (1)	53% (8)	
**Previous urethral surgeries**				.010
No previous surgery	37% (7)	0% (0)	47% (7)	
1 previous surgery	42% (8)	25% (1)	47% (7)	
More than 1 previous surgery	21% (4)	75% (3)	7% (1)	
**Previous urethroplasty**				.83
Yes	21% (4)	25% (1)	20% (3)	
No	79% (15)	75% (3)	80% (12)	
**Stricture length/cm **	3 (1.43)	3 (1.58)	2 (1.35)	.20
**Number of strictures**				.21
Single	53% (10)	25% (1)	60% (9)	
Multiple	47% (9)	75% (3)	40% (6)	
**Qmax**				
Qmax before surgery	5 (4.00-6.50)	4 (2.50-5.00)	6 (4.00-8.50)	.13
Qmax after surgery 3 M	32 (12.76)	17 (16.64)	35 (9.86)	.022
Qmax after surgery 6 M	32 (18.42)	26 (27.58)	34 (17.74)	.59
Qmax after surgery 12 M	24 (8.53)	9 (.)	26 (6.59)	.054
Qmax after surgery 24 M	20 (11.73)		20 (11.73)	
**Complications**				
Incontinence				
No	100% (19)	100% (4)	100% (15)	
Infection				
No	100% (19)	100% (4)	100% (15)	

## Data Availability

The data that support the findings of this study are available on request from the corresponding author.
